# Spontaneous Intracranial Hypotension and Multi-Level Cervical and Lumbar Epidural Blood Patches: A Case Report

**DOI:** 10.7759/cureus.27721

**Published:** 2022-08-05

**Authors:** André Parra, Flávia Relvas, Paulo M Pereira, Alexandre Carrilho

**Affiliations:** 1 Anesthesiology, Centro Hospitalar Universitário De Lisboa Central, Lisbon, PRT

**Keywords:** epidural space(ep), refractory headache, positional headache, intracranial hypotension syndrome, autologous blood patch, cerebrospinal fluid fistula, cerebrospinal fluid (csf)

## Abstract

Spontaneous intracranial hypotension (SIH) is a neurologic condition where the intracranial pressure is reduced due to a loss of cerebrospinal fluid from its reservoir, the intrathecal space, to surrounding tissues. It is commonly characterized by an incapacitating headache, phono-photophobia, nausea, and vomiting, commonly refractory to medical treatment and requires further investigation.

We describe the case of a healthy young man who presented to the emergency room with a postural headache, accompanied by nausea, vomiting, and phono-photophobia. Brain computed tomography (CT) imaging study was unremarkable and he was initially treated symptomatically. Because of persisting pain even on medical treatment, additional imaging studies, including a myelo-CT scan, were performed and a diagnosis of multi-level cerebrospinal fluid fistulas was made. To treat the underlying cause, a first epidural blood patch (EBP) was initially performed at C7-T1 with 20 mL of autologous blood, but failed to provide complete symptomatic relief. Months later, a second EBP was conducted at C6-C7 with higher volume (30 mL) but as in the first EBP this procedure too did not result in total resolution of the headache and accompanying symptoms. Since there was no surgical indication from Orthopedics and Neurosurgery and the symptoms persisted, a third EBP was carried out, this time at a lumbar level (L2-L3) with infusion of 60 mL of blood so the upper dorsal and cervical epidural space was reached. This resulted in a better symptom relief, allowing the patient to now carry out his normal activities with only residual pain.

The need for repeat procedures is one of the pitfalls of the blood patching technique. If possible, it should be performed at the level of the documented fistula, but always with safety in mind and by experienced hands, especially when cervical levels are concerned. A consensus has not been reached regarding the blood volume to be administered; however, any discomfort or pain reported by the patient should be seen as warning sign and the procedure should be interrupted. Although not being a perfect solution, EBP can completely or partially resolve SIH symptoms, without the need for surgical intervention.

## Introduction

Spontaneous intracranial hypotension (SIH) is a neurological condition, with an annual estimated incidence of five per 100 000, characterized by postural headache, associated with phono-photophobia, nausea, vomiting, and malaise [1,2]. Postural headache after cranial trauma, excessive physical exertion, or violent coughing should raise suspicion of SIH [1]. Treatment with conservative measures like bed rest, hydration, caffeine, and common analgesics is frequently short lasting and ultimately ineffective [3]. Since cervical liquor fistulas are not frequently documented, data remains scarce regarding its treatment and efficacy remains highly controversial [2].

This case report contributes to the current knowledge on more invasive strategies as treatment options for SIH, since there are very few cases on literature describing a cervical level approach.

## Case presentation

We present the case of a 34-year-old physically active and previously healthy, American Society of Anesthesiologists (ASA) I, Caucasian male, who presented to the ER with a two-month history of persistent postural headache that started while surfing. The day before the beginning of symptoms he had been practicing gymnastics on a trampoline, as he did on a regular basis. The pain was constrictive, non-pulsatile, rated 10 out of 10 on a numerical scale, was located to the occipital region, and radiated to both parietal regions. It subsided in supine position. Accompanying symptoms were nausea, vomiting, and phono-photophobia. He described the pain as an impairment to daily life activities.

Blood work-up and a computed tomography (CT) scan of the brain were unremarkable, and the patient was sent home on painkillers. After two months of persistent symptoms despite medical treatment, a cerebrospinal fluid (CSF) fistula was suspected and a neuro-axis magnetic resonance imaging (MRI) was performed. The MRI showed multiple cervical-dorsal collections of epidural CFS and a possible dural continuity solution at T2-T3 level.

An epidural blood patch (EBP) was performed at C7-T1 level, with the patient in right lateral recumbent (RLR) position. Using the loss of resistance technique, an 18G Tuohy needle with the bevel facing the patient’s right side was used to inject 20 mL of autologous blood. The patient was discharged with partial resolution of symptoms, but referring *de novo* lightheadedness.

**Figure 1:**
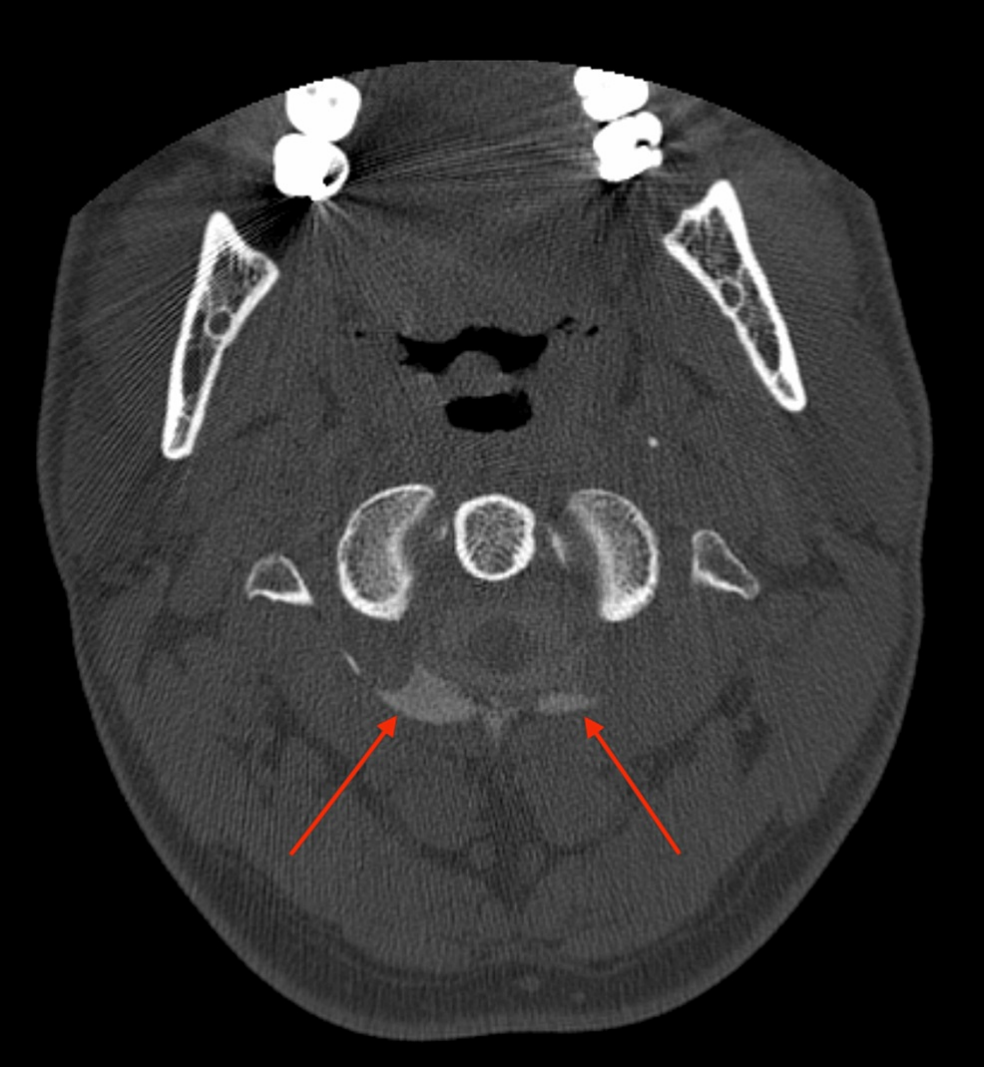
Axial C1-C2 level myelo-CT showing bilateral contrast outside the subarachnoid space (red arrows), as evidence of C1-C2 CSF fistulas.

**Figure 2:**
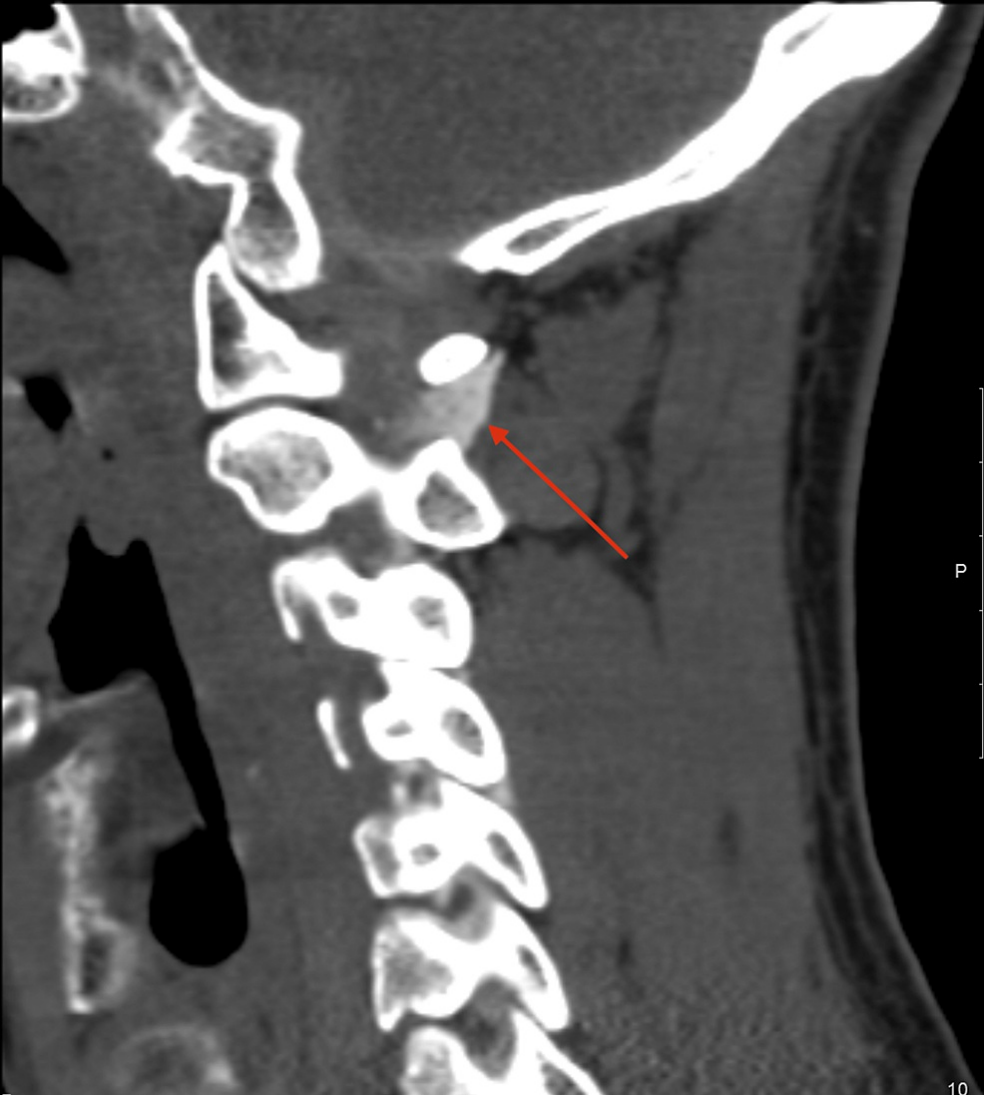
Parasagittal cervical myelo-CT showing contrast outside the subarachnoid space (red arrow), as evidence of a right-sided C1-C2 CSF fistula.

**Figure 3:**
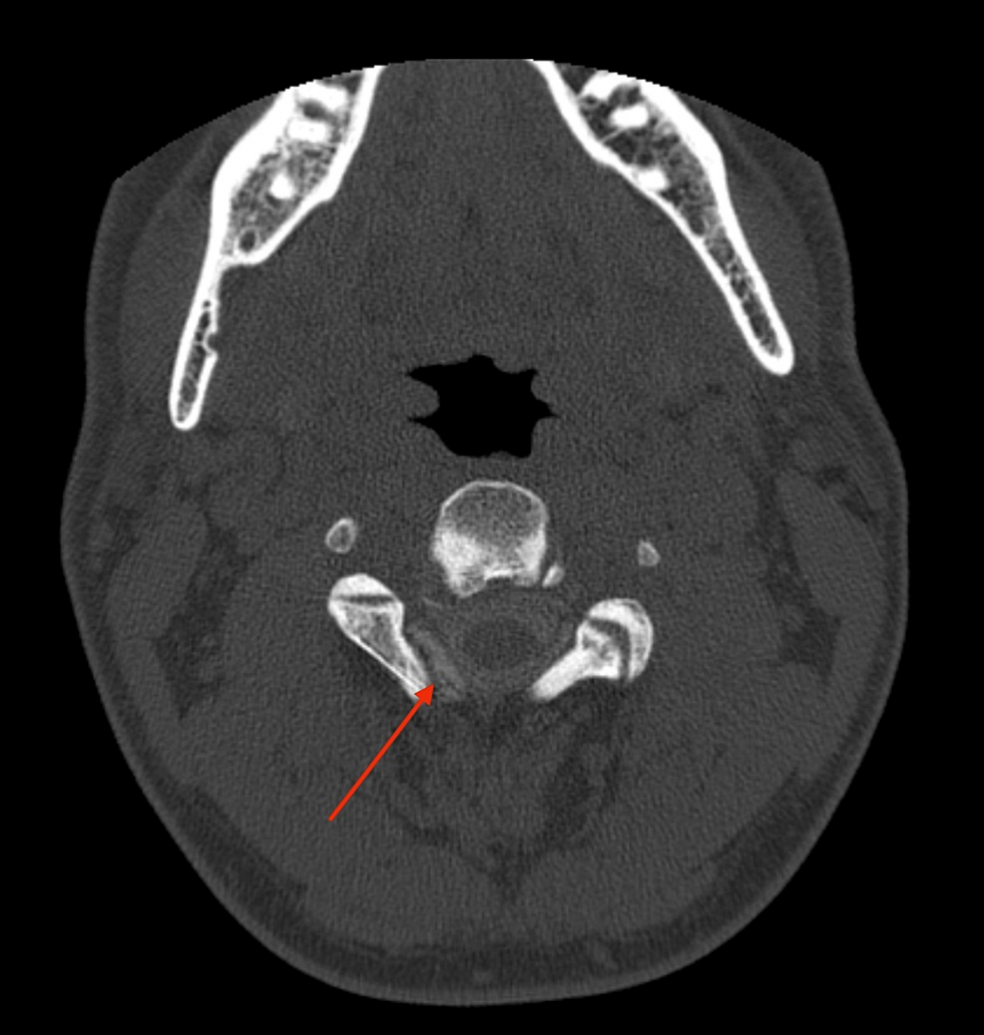
Axial C2-C3 level myelo-CT showing contrast outside the subarachnoid space (red arrow), as evidence of a right-sided C2-C3 CSF fistula.

**Figure 4:**
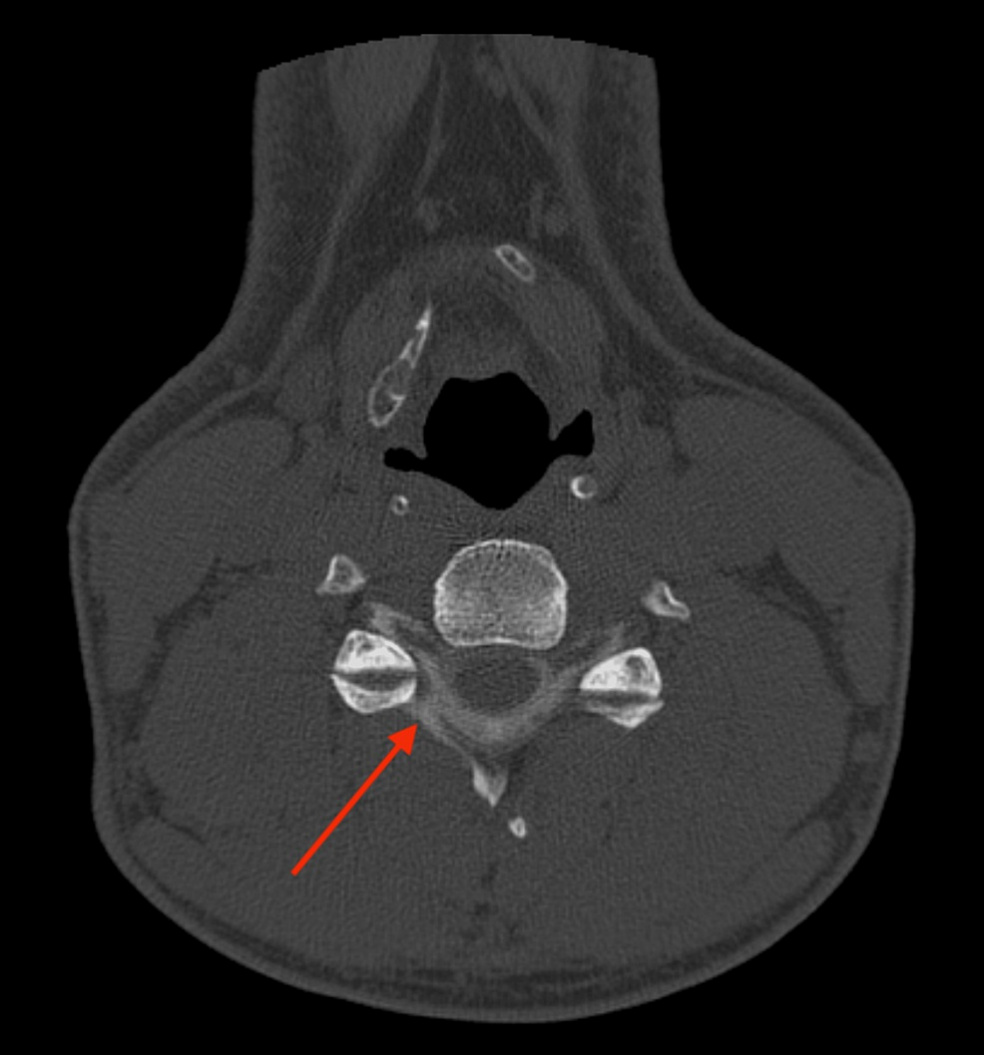
Axial C4-C5 level myelo-CT showing contrast outside the subarachnoid space (red arrow), as evidence of a right-sided C4-C5 CSF fistula.

After three months of persistent symptoms, a myelo-CT scan, with injection of radiographically opaque dye in the subarachnoid space, showed five cervical CSF fistulas: C1-C2 bilaterally (Figure 1 and Figure 2), C2-C3 right (Figure 3), C3-C4 left and C4-C5 right (Figure 4). A second cervical EBP was performed with 30 mL of autologous blood injected at C6-C7 level with a cranial-facing bevel. There was significant clinical improvement on the first day after procedure, and the patient described a less intense headache that worsened with mild efforts, particularly in the morning time, and was now unrelated to body positioning.

He was referred to Neurology and was started on gabapentin and amitriptyline, without any improvement in symptoms. After discussion with Orthopedics and Neurosurgery, there was no surgical indication.

Two months later, a third EBP was performed, this time at lumbar level L2-L3, again with the patient on RLR on a 15º angle Trendelenburg positioning. Bevel was facing upwards to obtain cranial dispersion. Through the loss of resistance technique, 60 mL of autologous blood was injected, and only partial improvement was achieved.

All three blood patches were safely conducted, with no adverse effects besides complaints of pressure on injection site. The initially planned blood volume was always injected in full. 

Since then, the patient started practicing yoga and received massages that alleviate the symptoms. A cervical pain characterized as a pressure-like pain (2 out of 10) persists to date and worsens by standing up rapidly or with physical strain (8 out of 10). It has an occipitotemporal bilateral irradiation and is also associated with photophobia. Nausea and vomiting subsided. A curious remark is the association between flares and periods of diminished water intake. When the pain is the most severe, the patient self-medicates with paracetamol, which improves the symptoms substantially.

## Discussion

Although blood patching is described as an effective treatment for orthostatic liquor hypotension in the context of cervical CSF fistulas, this clinical case demonstrates the possibility of repeat-procedures at different spinal levels in order to achieve symptomatic relief and even then, a non-complete resolution of symptoms [4]. More invasive techniques, such as surgery, may be needed in fistulas documented on neuroimaging.

One of the main risks of this blood patch procedure is puncturing the dura mater and reaching the subarachnoid space, possibly harming any structures in it. As it is known, in adults the spinal cord ends at the beginning of the lumbar vertebrae, so needle punctures below this level are safer since only the cauda equina is present in the subarachnoid space. In this particular case, the first approach could have been a lumbar EBP, as it is also easier to execute, but given the location of the documented fistulas a cervical approach was preferred. Here, the epidural space is much narrower than at the lumbar levels, so the volume injected must accordingly be lesser.

This poses an important question: what is the adequate volume of blood to infuse? The answer must take into account several factors, namely the injection site level, speed of injection, and tolerability. Several case reports describe small volumes for the cervical approach (5-8 mL) [4] compared to the volume of our cervical blood patches (20 and 30 mL), all without major safety concerns. The speed of injection can modify the volume of blood since a slow and steady procedure allows for the epidural space to be broadened out gently without major symptoms. These are dependent on the EBP level and may include spinal pressure, numbness of a specific territory (arm, leg), or even acute pain, all of which should be seen as warning signs and prompt the question whether to continue or stop the procedure.

Patient positioning may also be a variable to take into account. Nonetheless it is of major importance that the operator is comfortable and proficient, since supine and lateral recumbent positions entrain different directions of needle movement that one might not be used to. This premise of proficiency is also applied to the identification of the epidural space, either through the loss of resistance or the hanging drop technique. In our case due to operator experience, the patient was in a lateral recumbent position and the loss of resistance technique was preferred.

Discussion must also include the type of liquid injected. Nowadays, most commonly, the technique is done through the injection of autologous fresh whole blood drawn from the patient at the same time of the procedure, but some advocate the use of platelet-rich plasma (PRP), in either case aiming to regenerate and heal the damage tissue where the CSF fistula occurs [5]. Both options act by two different mechanisms: the first and most immediate, the increase of pressure in the epidural space is transmitted to the subarachnoid space and, because the intrathecal hypotension is being opposed, a rapid sense of symptom relive is achieved. The second and long-lasting mechanism, the clotting factors present in both whole blood and PRP will create a seal that prevents CSF from leaving the subarachnoid space, hence maintaining epidural integrity. Other options to be considered include continuous epidural saline infusion, fibrin glue, and, ultimately, surgical repair.

## Conclusions

Our report shows that careful and timely spaced procedures, although not entirely successful, were able to ameliorate the quality of life of our patient. Having documented the CSF fistulas' levels, a tailored approach was paramount. EBPs are commonly performed in the obstetric setting, at a lumbar level. A cervical EBP is an uncommon technique, mainly due to its potential neurological complications. Side effects can be minimized when taking into account several precautions, such as spine level, blood volume, patient positioning, and technique when identifying the epidural space. Nonetheless, it seems to be a safe procedure in skilled hands.

Given the questions surrounding multiple variables when performing an EBP, more studies are needed to evaluate its formal indications, indications for a multi-level approach, time between procedures, ideal volume of injected blood, best blood product, and overall efficacy and effectiveness.
